# Recent increased identification and transmission of HIV-1 unique recombinant forms in Sweden

**DOI:** 10.1038/s41598-017-06860-2

**Published:** 2017-07-25

**Authors:** Ujjwal Neogi, Abu Bakar Siddik, Prabhav Kalaghatgi, Magnus Gisslén, Göran Bratt, Gaetano Marrone, Anders Sönnerborg

**Affiliations:** 1 Division of Clinical Microbiology, Department of Laboratory Medicine, Karolinska Institutet, Huddinge, Stockholm Sweden; 2 Department of Computational Biology and Applied Algorithmics, Max Planck Institute for Informatics, Saarbrücken, Germany; 3 Department of Infectious Diseases, Sahlgrenska Academy, University of Gothenburg, Gothenburg, Sweden; 4 Department of Infectious Diseases, South Hospital, Stockholm, Sweden; 5 Department of Public Health Sciences, Karolinska Institutet, Solna, Sweden; 6 Department of Medicine Huddinge, Unit of Infectious Diseases, Karolinska Institutet, Karolinska University Hospital, Stockholm, Sweden; 7 Present Address: Medical Microbiology Department, University of Manitoba, 727 McDermot Ave, Winnipeg, MB R3E 3P5 Canada

## Abstract

A temporal increase in non-B subtypes has earlier been described in Sweden by us and we hypothesized that this increased viral heterogeneity may become a hotspot for the development of more complex and unique recombinant forms (URFs) if the epidemics converge. In the present study, we performed subtyping using four automated tools and phylogenetic analysis by RAxML of *pol* gene sequences (n = 5246) and HIV-1 near full-length genome (HIV-NFLG) sequences (n = 104). A CD4^+^ T-cell decline trajectory algorithm was used to estimate time of HIV infection. Transmission clusters were identified using the family-joining method. The analysis of HIV-NFLG and *pol* gene described 10.6% (11/104) and 2.6% (137/5246) of the strains as URFs, respectively. An increasing trend of URFs was observed in recent years by both approaches (p = 0·0082; p < 0·0001). Transmission cluster analysis using the *pol* gene of all URFs identified 14 clusters with two to eight sequences. Larger transmission clusters of URFs (BF1 and 01B) were observed among MSM who mostly were sero-diagnosed in recent time. Understanding the increased appearance and transmission of URFs in recent years could have importance for public health interventions and the use of HIV-NFLG would provide better statistical support for such assessments.

## Introduction

Description of regional epidemics of the human immunodeficiency virus type 1 (HIV-1) is facilitated by the large number of *pol* sequences generated for genotypic drug resistance testing (GRT) in clinical care. Using such sequences, we have earlier reported that all known subtypes and circulating recombinant forms (CRFs) are present in Sweden, to a large extent as a consequence of migration from high endemic African and Asian countries^1, 2^. It has transformed the Swedish HIV-1 epidemic to one of the most diverse epidemics outside Africa^1^. We hypothesized that this viral heterogeneity may become a hotspot for the development and spread of more complex and unique recombinant forms (URFs). The identification of such recombinants is enhanced by near full-length genome sequencing of HIV (HIV-NFLG)^3, 4^.

The majority of people living with HIV in Sweden are migrants infected by various subtypes^5^. Several subtypes are also circulating among people who inject drugs (PWID)^6^ but among men who have sex with men (MSM) HIV-1 subtype B (HIV-1B) is reported to be still predominant^6^. However this information is based on analysis of smaller gene fragments^1, 2, 6, 7^. When two or three genes are included in determining the subtype, the identification of inter-subtype recombinants increases significantly^8, 9^. The use of HIV-NFLG improves further the understanding of the dynamics of the pandemic at the population level^10^, clustering statistics^11^, viral diversification^12^ as well as the identification of drug resistance mutations to all drug classes^10, 12^. As of 2015, only 16 HIV-NFLG sequences have been reported from Sweden of which seven were URFs, exclusively found in migrants from Africa^13–16^.

The aim of the present study was to investigate the distribution and transmission of HIV-1 subtypes and recombinant forms in Sweden using HIV-NFLG sequences derived from archival plasma specimens sampled during the last two decades. To our knowledge this is the first study in a European country where a large number of HIV-NFLG have been used to describe the molecular epidemiology in a specific country. Also, we used *pol* gene sequences obtained in clinical care from the national InfCare HIV database, which covers >99.9% of living patients, in order to give an overall picture of the appearance of URFs.

## Results

### Clinical characteristics of the patients with HIV-NFLG

An HIV-NFLG was obtained in 104 out of 148 (70.3%) tested samples (Table 1). There was no significant difference with regard to demographic or biomedical data between the patients in whom NFLG failed or not. According to reported country of transmission, 43% of the patients (n = 45) were infected in and 56% patients outside (n = 59) Sweden, with no information for 10 (1%) patients. The duration of the HIV infection was estimated in 88 (77%) patients through either the CD4^+^ T-cell decline trajectory model (n = 79) (Supplementary Table S1) or a serologically verified primary HIV infection (PHI) (n = 9). The patients were then categorized into those diagnosed before 2005 (n = 48), between 2005 to 2010 (n = 26), and those after 2010 (n = 14).

**Table 1:** Patient’s characteristics in whom near full-length HIV-1 genome sequencing was performed.

	Country of transmission*		p
	Sweden (n = 45)	Outside (n = 59)	
Age in years; median (IQR)	44 (35–52)	36 (30–41)	<0·0001
Gender; Female n (%)	14 (31)	34 (57)	0·0098
Route of transmission; n (%)			
Heterosexual	24 (53)	47 (80)	0·0005
MSM	12 (27)	7 (12)	
PWID	8 (18)	0	
Other/Unknown	1 (2)	5 (8)	
HIV-1 RNA load; log_10_ copies/mL	5·08 (4·51–5·17)	5·19 (4·69–5·7)	0·9823
CD4 count cells/μl; median (IQR)	240 (150–370)	204 (101–350)	0·3982
HIV-1 subtyping; *pol*; n (%)**			
A1	2 (4)	3 (5)	0·0083
B	17 (34)	6 (10)	
C	19 (38)	39 (66)	
D	0	1 (2)	
01_AE	4 (8)	3 (5)	
02_AG	0	5 (8)	
Other CRFs	0	1 (2)	
URFs	3 (6)	1 (2)	

MSM: men who have sex with men; PWID: people with intravenous drug use; CRFs: circulating recombinant forms; URFs: unique recombinant forms; *reported by the treating physician; **based on the *pol* gene.

### Distribution of subtypes, CRFs and URFs at NFLG analysis

**Figure 1:**
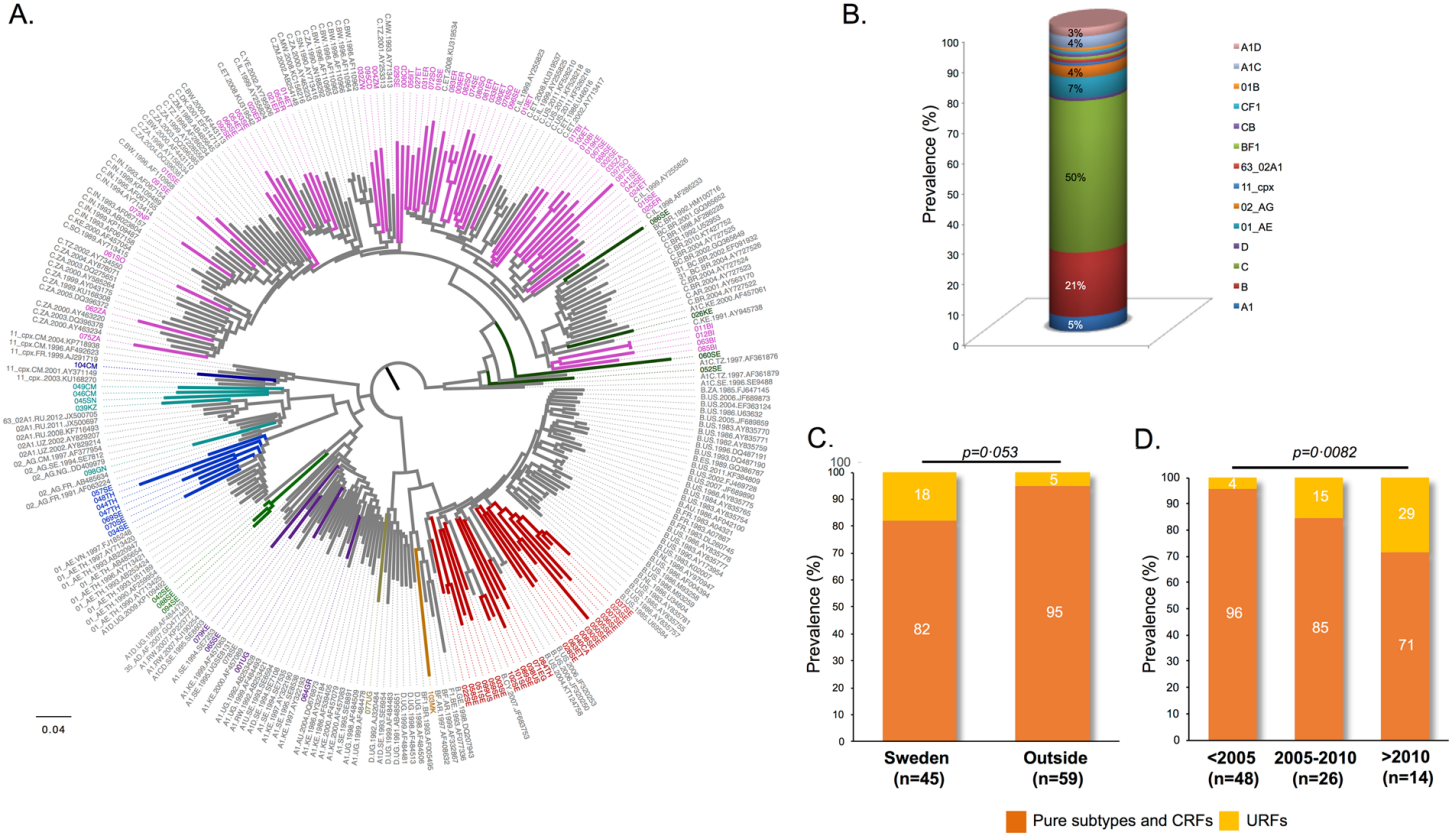
(**A**) Maximum-likelihood phylogenetic analysis. The HIV-NFLG sequenced in our study (n = 104) and a unique set of 175 reference sequences downloaded from Los Alamos database were used. (**B**) Distribution of HIV-1 pure subtypes (n = 80), circulating recombinant forms (CRFs) (n = 13) and unique recombinant forms (URFs) (n = 11) based on three subtyping tools (REGA v3, RIP 3.0 and COMET-HIV) and ML-phylogenetic analysis. (**C**) Proportion of pure subtypes/CRFs and URFs among patients infected in or outside Sweden, according the reports from the treating physician. A higher proportion of URFs was identified among patients infected in Sweden (n = 8; 18%) than outside (n = 3; 5%) (Fisher’s exact test p = 0·0523). (**D**) Distribution of pure subtypes/CRFs and URFs in relation to year of transmission predicted by the CD4^+^ T-cell decline trajectory algorithm (n = 79) or by a serologically verified primary HIV infection (n = 9). The trends indicate a significant increase of URFs in newly diagnosed patients in Sweden over time (Chi-square test: 6·986; p = 0·0081).

The 104 NFLG were analyzed by the three automated subtyping tools and the ML-phylogenetics (Fig. 1A). Most strains (77%; 80/104) were pure subtypes (C: n = 52; B: n = 22; A1: n = 5; D: n = 1) (Fig. 1B). Recombinant forms accounted for 23% (24/104) with 13 (12.5%) CRFs (01_AE: n = 7; 02_AG: n = 4; 11_cpx: n = 1; 63_02A1: n = 1) and eleven URFs (10.5%) (A1C: n = 4; A1D: n = 3; 01B: n = 1; BF1: n = 1; CF1: n = 1; BC: n = 1) (Fig. 1B).

A higher proportion of URFs was identified among patients reported to be infected in Sweden (n = 8; 18%) than outside the country (n = 3; 5%) (p = 0·0530) (Fig. 1C). Based on the patients who had an estimated time of infection (n = 88), there was a significant increase of URFs in recent years (p = 0·0082) (Fig. 1D).

### Detailed characterization of unique recombinant forms

**Figure 2:**
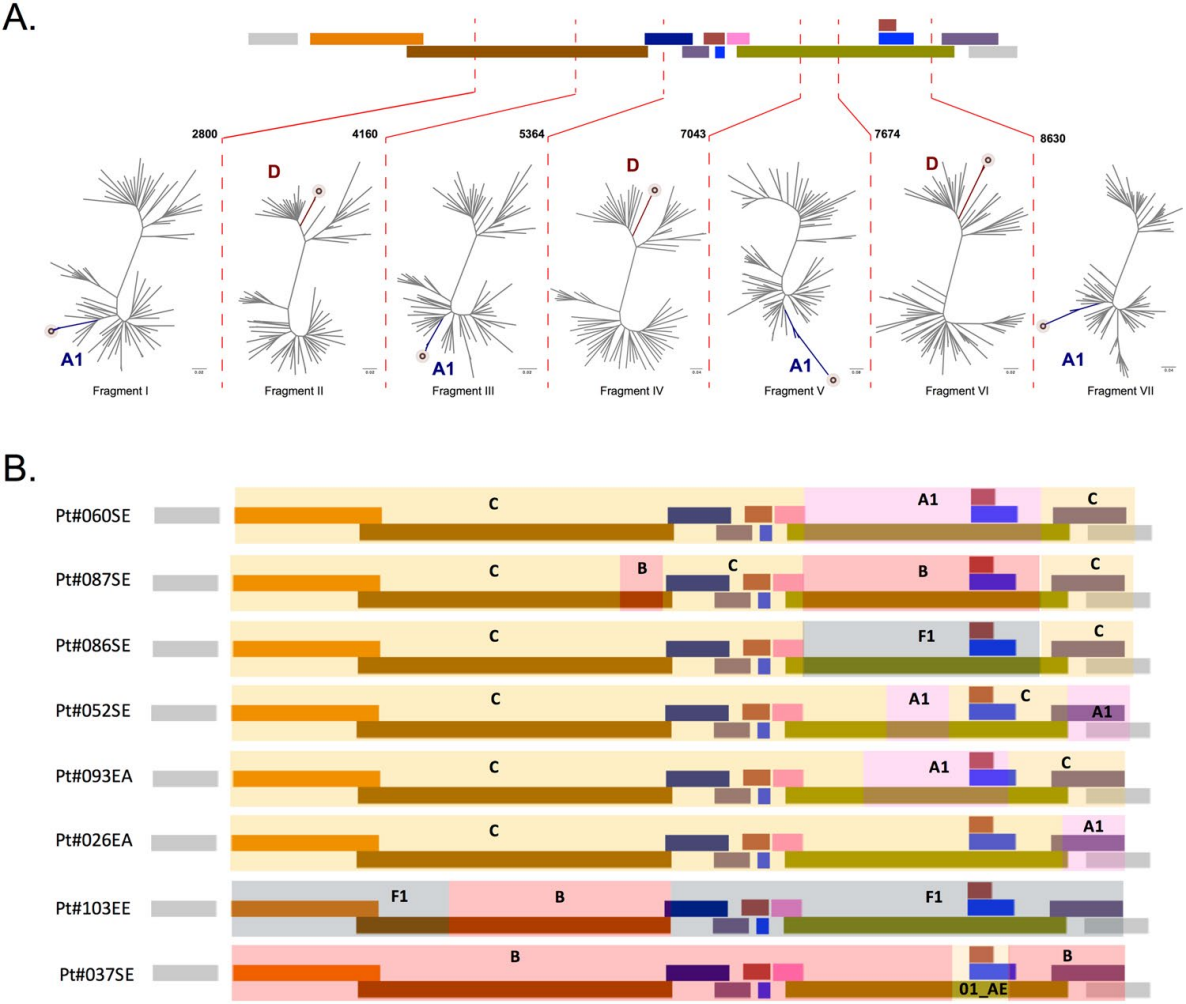
(**A**) Mosaic pattern of three HIV-1 A1D recombinants. Precise inter-subtype recombination analysis was performed using bootscanning analysis and similarity plot analysis implemented in SimPlot ver. 3.5.1 with 500 bp window size and 20 bp step size^36^, Recombination Detection Program (RDP) ver.4^37^ and jumping profile Hidden Markov Model (jpHMM)^38^. The recombination breakpoints are indicated as an HXB2 position. ML-phylogenetic analysis was used to confirm the recombination events. Patients were self-reported to be men who have sex with men and infected in Sweden. (**B**) Mosaic pattern of eight other HIV-1 URFs. These URFs did not belong to any identified transmission cluster. SE: patient infected in Sweden; EE: patient infected in East-Europe; EA: patient infected in East Africa.

We characterized the recombinant forms in detail using SimPlot ver3.5.1, RDP ver4 and jpHMM, followed by fragment specific ML-phylogenetic analysis. The three URF_A1D, obtained from heterosexuals infected in Sweden, shared a nearly similar mosaic structure (Fig. 2A). Fragments I (HXB2: 790–2800), III (HXB2: 4161–5364), V (HXB2: 7044–7674) and VII (HXB2: 8630–9555) were of HIV-1A1 while fragments II (HXB2: 2801–4160); IV (HXB2: 5365–7043) and VI (HXB2: 7674–8630) were of HIV-1D. The fragments V and VII were more diverse than the other fragments. In contrast, the four URF_A1C, obtained from two patients infected in Sweden and two patients infected in Eastern Africa, had a different mosaic pattern and were not directly related (Fig. 2B).

When only the *pol* genes of the eleven URFs, identified by HIV-NFLG, were analysed, four were classified as URFs (A1D: n = 3; BF1: n = 1), while seven (A1C (n = 4), BC, 01B and CF1) were identified by HIV-NFLG only. Of these latter seven URFs, three were (01B, BC, and CF1) identified in MSM who had been infected in Sweden.

### Clinical characteristics of patients with URFs identified by *pol* sequencing

Subsequently, we analyzing the *pol* genes from the complete Swedish InfCare HIV database (n = 5246), 137 URFs (2.6%) were identified. The majority of the patients infected with URFs were sampled from heterosexuals (54%, 74/137) followed by MSM (27%; 37/137), unknown/other (12%; 17/137), mother to child transmission (4%; 5/137), through blood transfusion and PWID (3%; 4/137). Almost half of the patients (46%; 63/137) diagnosed with a URF had been infected ≥5 years at diagnosis, as determined by the CD4^+^ T-cell trajectory model, with a median CD4^+^ T-cell count of 188 cells/μl (IQR: 88 to 231) at diagnosis. This is to be noted that 97% (36/37) of the URFs identified among the MSM had HIV-1B as one of the fragment in the recombinant forms.

A similar trend of increasing appearance of URFs with time as for the NFLG analysis was observed for the 137 patients infected by URFs, as determined by *pol* gene analysis. Thus, the proportion of URFs among the samples obtained before 2005 was 1%, which increased to 4% after 2010 (Chi-square test for trend 12·57, p < 0·0001).

### Transmission clusters identified by HIV-NFLG

**Figure 3:**
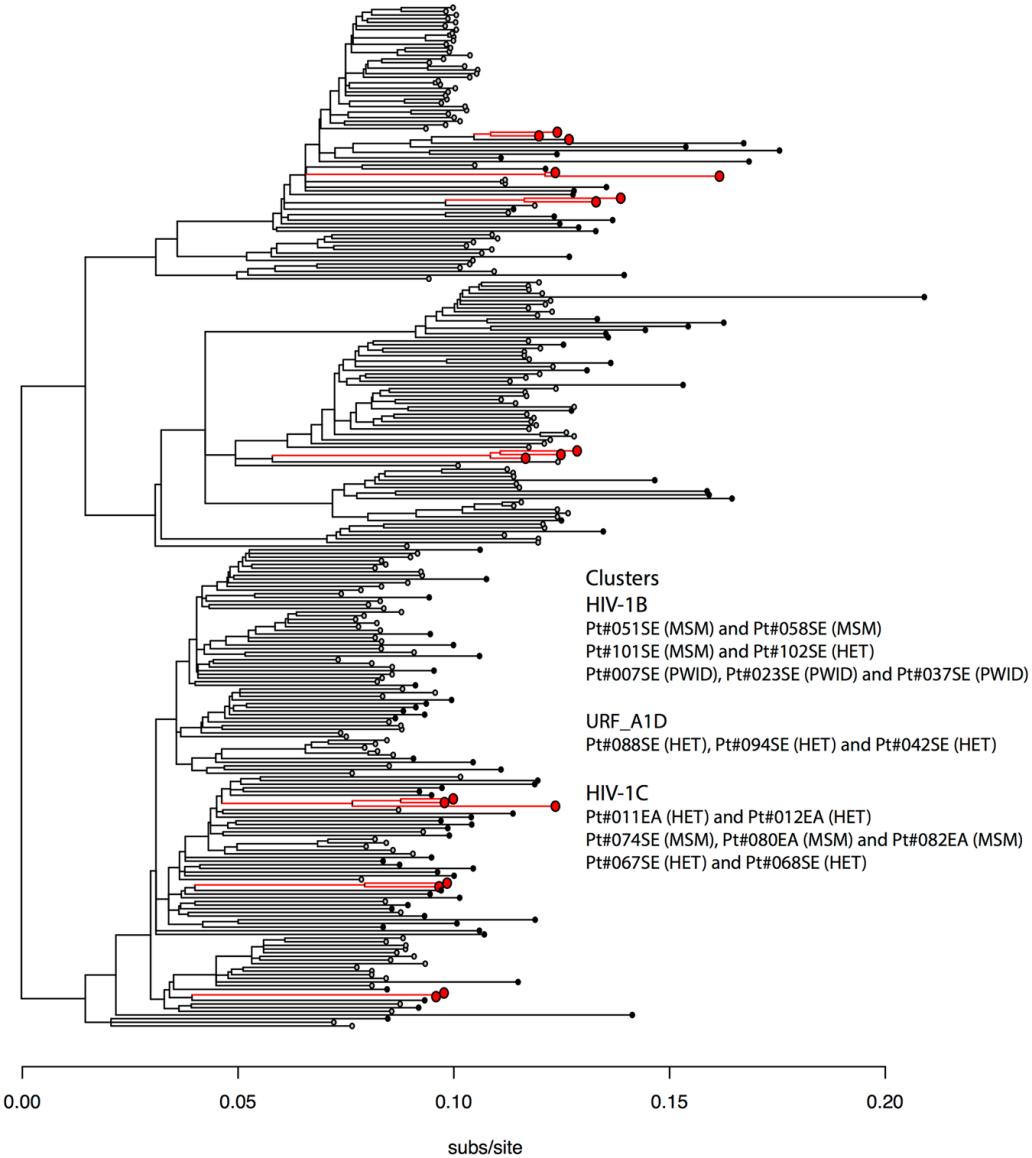
The evolutionary relationships based on HIV-NFLG using family-joining. Transmission clusters (n = 7) were constructed for trees at a threshold of 0·08 substitutions per site and are indicated within the figure with red colour. SE: patient reported to have been infected in Sweden; EA: patient reported to have been infected in East Africa. MSM: men who have sex with men; HET: patient self-reported to have been infected heterosexually; PWID: patient with intravenous drug use.

Subsequently we inferred evolutionary relationships using family-joining. Transmission clusters were constructed for trees based on the HIV-NFLG, at thresholds of 0.08 substitution/site (Supplementary Fig. S2). The C_NFLG tree identified seven clusters, consisting of HIV-1B (n = 3), HIV-1C (n = 3) and URF_A1D (n = 1), respectively (Fig. 3). All three HIV-1B transmission clusters (cluster 1: two MSM; cluster 2: one MSM and one heterosexual; cluster 3: three PWID) and one HIV-1C transmission cluster (two heterosexuals) had occurred within Sweden. For URF_A1D all three individuals had been infected heterosexually in the country. In one HIV-1C cluster with three transmission events, there were one Swedish MSM and two individuals originating from a Sub-Sahara African country, who were reported to be heterosexual and MSM, respectively. In the two remaining HIV-1C clusters, two heterosexuals had been infected in a central-African country and two heterosexuals in Sweden, respectively.

Transmission clusters were also constructed for trees based on the *pol* sequences obtained through NFLG at thresholds of 0.02 substitution/site. The C_NFLG and C_pol trees had six clusters in common. The Jaccard index of the two sets of clusters was thus 0.67 (6/9). In one HIV-1C cluster, C_NFLG included three patients’ sequences (Pt#074, 080 and 082) of which two were there described in the *pol* (Pt#074 and 080) gene analysis. One additional cluster was found in C_pol only (Pt#005 and 030) which was absent in C_NFLG. This data indicates false identification of clusters in the *pol* analysis due to low statistical power.

### Transmission clusters identified by *pol* analysis

Transmission clusters were also constructed for trees based on the 137 URFs obtained through analysis of routine *pol* sequences at thresholds of 0.02 substitution/site (Supplementary Fig. S2). A total of 14 clusters consisting of two to eight sequences were observed. All six of the two sequence clusters (one each A1C, A1B, 02A1, BC, and two different 02A1) were observed among 12 heterosexuals and all but one of these small clusters had appeared outside Sweden. Among MSM (n = 37), five clusters with three to eight patients were observed (two clusters of BC; one each of 01D, BF1, and 02B), which comprised 27 of the 37 (73%) men.

Eight larger URFs transmission clusters were identified, consisting of three to eight individuals (Table 2). The largest of these clusters (BF1: n = 8 individuals; 01B: n = 7 individuals) were observed among MSM. Three clusters of one each URFs DC (n = 4 patients), A1D (n = 3 patients) and BF1 (n = 3 patients) were observed among the individuals who had been infected through a heterosexual (n = 8) or other/unknown (n = 2) route.

**Table 2:** Characteristics of eight transmission clusters with more than two patients, based on the *pol* gene using the FJ-method.

Cluster	ID	Country of transmission*	Route of transmission*	Year of diagnosis	CD4**	Estimated year of infection. Median (IQR)
URF BC	087	Outside	MSM	2012	450	2010 (2008–2012)
	112	Sweden	MSM	2014	430	2011 (2008–2013)
	093	Outside	MSM	2013	580	2012 (2010–2012)
	111	Sweden	MSM	2014	580	2013 (2011–2013)
URF BF1	**136**	Sweden	MSM	2016	220	2009 (2007–2011)
	**127**	Outside	MSM	2015	260	2009 (2007–2011)
	**128**	Outside	MSM	2015	305	2010 (2008–2012)
	114	Outside	MSM	2014	460	2012 (2009–2013)
	129	Sweden	MSM	2015	400	2012 (2010–2014)
	135	Outside	MSM	2015	440	2013 (2011–2015)
	125	Sweden	MSM	2014	728	2014 (2013–2014)
	132	Outside	MSM	2015	550	2014 (2012–2015)
URF 01B	**050**	Sweden	MSM	2009	64	1997 (1994–1999)
	**077**	Sweden	MSM	2011	66	2000 (1997–2002)
	**055**	Sweden	MSM	2009	218	2002 (1999–2004)
	046	Sweden	MSM	2009	624	2008 (2007–2009)
	054	Sweden	MSM	2009	304	2009 (PHI)***
	098	Sweden	MSM	2012	330	2009 (2006–2011)
	092	Sweden	MSM	2013	360	2009 (2007–2011)
URF BC	**027**	Sweden	MSM	2008	216	2002 (2000–2004)
	**086**	Sweden	MSM	2012	130	2004 (2002–2006)
	066	Outside	MSM	2010	461	2008 (2006–2010)
	056	Sweden	MSM	2009	1480	2009 (2008–2009)
URF 02B	084	Sweden	MSM	2012	400	2009 (2007–2011)
	083	Sweden	MSM	2012	510	2010 (2008–2011)
	121	Sweden	MSM	2014	500	2013 (2010–2014)
	133	Outside	MSM	2015	480	2014 (2011–2015)
URF DC	**035**	Outside	HET	2005	<10	1991 (1989–1993)
	**008**	Outside	OTH	2004	202	1997 (1993–1999)
	**010**	Sweden	HET	2006	102	1998 (1997–2000)
	**049**	Sweden	HET	2009	311	2004 (2001–2006)
URFA1D	**063**	Sweden	HET	2010	196	2004 (2002–2006)
	**102**	Sweden	HET	2013	80	2005 (2003–2007)
	107	Sweden	HET	2013	431	2011 (2009–2013)
URF BF1	**021**	Outside	HET	2002	80	1991 (1988–1993)
	**030**	Sweden	HET	2003	170	1997 (1995–1999)
	024	Sweden	OTH	2005	315	2001 (1999–2003)

Transmission clusters were constructed at 0.02-subs/site thresholds. *Reported by the treating physician; **cells/μl at HIV-1 diagnosis; ***PHI: primary HIV infection; MSM: men who have sex with men; Hetero: heterosexually infected; OTH: other mode of transmission or unknown; Late presenters are marked in bold.

For 16 out of the 37 patients who belonged to six out of the eight large clusters, the time of HIV-1 transmission was estimated to be at least five years earlier (median: 7 years, range: 5–14) than the time of diagnosis (Table 2). Also, all of them had a CD4^+^ T-cell count below 350/μl at diagnosis, fulfilling the criteria of being a late presenter^17^. In three clusters including heterosexually infected individuals, the three out of ten persons estimated to have been infected for the longest time were reported to have been infected outside Sweden, the remaining seven were infected in Sweden. For MSM, a more mixed pattern was seen with nine out of the 27 (33%) subjects reported to have been infected outside Sweden.

## Discussion

In the present study, the appearance and spread of transmission clusters of unique recombinant forms (URFs) in Sweden over time was investigated using near full-length HIV-1 genomes (HIV-NFLG) and *pol* gene sequences. By both approaches, we identified an increased incidence of URFs among individuals diagnosed in the country and transmission of such strains within the country, in recent years. Several cases of cross-transmissions between MSM and persons who have a self-identity as heterosexuals were also identified. Our data indicates that intermixing of strains may occur within the country with a potential for the development of more complex recombinant forms as well as further spreading of such URFs.

Based on the *pol* gene, multiple subtypes have been reported in Sweden from the beginning of the HIV epidemic^2^. Several subtypes have been introduced among PWID^6^ and heterosexuals^1^, but in MSM HIV-1B is still reported to be predominant^1^. However, analysing only one gene fragment underestimates the presence of true recombinant forms^8–11^, which was confirmed in our study. Thus, using HIV-NFLG, a more accurate description of URFs in the Swedish HIV-1 epidemic can be obtained. It is therefore likely that the incidence of URFs in Sweden was underestimated in our analysis of the whole database and that more than 4% of newly diagnosed patients are infected with URFs after 2010. A similar situation may be present in other European countries. Based on *pol* gene analysis, increases of HIV-1A1 and HIV-1C have thus been reported among MSM in United Kingdom^18^ and Greece^19^. It is possible that an even higher proportion of recombinant forms had been identified in these studies if NFLG instead had been used.

The subtype distribution among MSM in Sweden between 1983 and 2012 was still dominated by HIV-1B, based on our earlier analysis of the *pol* gene^1^. In the present study, a significant trend was observed with a higher proportion of URFs among patients who were diagnosed the recent years, both when analyzing the HIV-NFLG or only the *pol* gene, supporting the notion of an increased viral heterogeneity in the Swedish HIV-1 epidemic. Interestingly, almost all (97%) of the URFs detected among MSM had an HIV-1B gene fragment, including the two major clusters, URF_01B and URF_BF1. As the epidemic among the MSM has been dominated by HIV-1B in the past, it is likely that this is a consequence of a more recent introduction of non-B subtypes among the MSM population. Actually, all larger URFs clusters (four to eight individuals) were observed among MSM, while small clusters of two sequences were mainly restricted to heterosexuals. It shall be noted that transmissions of URFs_01B in China^20^ and URF_BF1 in Brazil^21^ have been reported among MSM, although these URFs are different than those observed in our cohort.

We used HIV-NFLG to determine the clustering pattern using the newly developed FJ-method^22^ and compared with the use of the *pol* gene only. Our data indicated that *pol* gene analysis may overestimate clustering statistics, as earlier described. The HIV-NFLG clustering analysis observed two clusters (one each HIV-1B and HIV-1C) where there were cross-transmission events between persons reported to be heterosexually infected and MSM. All of these heterosexuals were black-African men who are more likely to self-identify themselves as heterosexuals compared with other ethnicities^23^. This is in line with a recent study which reported multiple occasions of shared transmission clusters between MSM and heterosexuals in the Nordic countries^24^. Also, a large study from United Kingdom, based on the *pol* gene, reported crossover transmission of HIV-1C from heterosexuals to MSM, which has led to an expansion of this subtype in United Kingdom^25^. Our phylogenetic analysis of the *pol* gene obtained from the large national database indicated that major transmission clusters were restricted to the MSM population with no crossover transmissions identified, indicating an added value of NFLG for understanding the HIV-1 epidemic.

In the Swedish setting, deficiencies in the health care system with missed HIV testing opportunities contribute to a high proportion of late presenters in whom the time of transmission often is unknown^26^. We used a CD4^+^ T-cell decline trajectory model to deduce the predicted year of actual infection and observed increasing trends of URFs in patients infected recent years. Also, URFs were diagnosed more frequently among patients reported to be infected in Sweden. The CD4^+^ T-cell trajectory model was originally designed to be used mainly on a population level. In this study, it was used on an individual level and this may have introduced bias in our estimation. However it should be noticed that when using the CD4^+^ T-cell trajectory model at an individual level, it has been shown to give valid and robust estimates, when compared with data obtained from physicians and phylogenetic analysis^27^. Our analysis showed that a substantial proportion of the patients with URFs had been infected for more than five years and had an advanced immunodeficiency at diagnosis. Thus, the failure of diagnosing these patients at an earlier stage of infection has contributed to the spread of URFs in Sweden.

In conclusion, our study provides molecular evidence of a higher detection rate of URFs by HIV-NFLG compared to analysis of *pol* gene fragment in an epidemic where diverse subtypes are circulating. Transmission of the URFs seems to have increased in recent years among the MSM infected in Sweden, partly as a result of amalgamating with migrants. As molecular surveillance with NFLG provides greater statistical support for clustering, HIV-NFLG sequencing of newly diagnosed cases within a country is likely to promptly detect changes in the viral genetic composition of the epidemic. This could contribute to a better understanding of HIV-1 transmission networks and potential of improved public health interventions in countries like Sweden as well as other countries where multiple subtypes are present.

## Material and Methods

### Clinical Specimens

Two categories of sequences were analysed: i) HIV-NFLG: attempts were done on archived plasma of 148 participants included in the Swedish InfCare HIV cohort, drawn over a time of 22 years (1993 to 2016) (Table 1)^28^. Whether transmission had occurred in or outside Sweden was reported by the treating physician, based on the interview with the patient. The selection of the patients was done randomly, based on the availability of stored plasma, and aiming at similar proportions of reported transmissions in or outside Sweden distributed over the chosen time period; ii) pol *genes*: the complete Swedish InfCare HIV database, including 5246 *pol* sequences from unique patients, was downloaded on 06 June 2016. The sequences had been obtained through routine GRT performed during the same time-period (1993 to 2016). The database includes > 99.9% of diagnosed living HIV diagnosed patients in Sweden, the majority of patients ever diagnosed in Sweden (n = 10738), and almost all routine *pol* sequences ever performed^1^. The Swedish HIV treatment guidelines have recommended GRT in all newly diagnosed patients since 2003^29^. The coverage rate has been around 60% since 2000 and has the recent years increased from 64% in 2010 to 84% in 2016 (median 71%). GRT has also been performed on patients failing ART since the middle of the 1990-thies, at the larger HIV clinics^30^.

### CD4^+^ T-cell decline trajectory model for estimation time of infection

In addition to self-reported time of infection, we also used a CD4^+^ T-cell decline trajectory algorithm to estimate time of HIV-1 transmission, after having identified and adjusted for factors associated with the slope of decline among identified groups of HIV-1 seroconverters (age and region of birth), as described by us recently^27^. The time of estimated HIV seroconversion was presented in three estimates; the earliest probable time of seroconversion, the median probable time, and the latest probable time. We did not apply the CD4 trajectory model to serologically verified PHI. Actual date of serology is presented as time (year) of infection.

### HIV-1 near full-length genome sequencing (HIV-NFLG)

Viral RNA was extracted using the QIAamp Viral RNA Extraction Kit, Qiagen, Germany, as per manufactures instructions. The NFLG amplified the 9 kb HIV-1 genome in two fragments followed by sequencing by two approaches: Sanger sequencing using 17 sequencing primers^13^ or next generation sequencing (NGS) in Illumina HiSeq. 2500, followed by consensus sequence generation using *in-house* bioinformatics pipeline, as recently described by us^31^. The NGS was validated against an external quality control (EQC) panel. Clustering of the consensus sequences generated by NGS and Sanger sequencing from a given sample was identified by maximum likelihood phylogenetic analysis with 100% bootstrap support and both type of sequences could thus be used simultaneously in the molecular epidemiology studies^31^.

### HIV-1 subtyping and identification of recombination

Reference HIV-NFLG sequences were downloaded from the Los Alamos (LANL) database. All HIV-NFLG sequences generated were submitted to the BLAST tool available in the LANL database. A unique set of 175 reference sequences were used for phylogenetic analysis as well as cluster analysis. HIV-1 subtyping were carried out using REGA v3^32^, Recombination Identification Program (RIP) v3^33^ and COMET-HIV^34^ followed by maximum likelihood phylogenetic tree using RAxML^35^. Precise inter-subtype recombination analysis was performed by bootscanning analysis and similarity plot analysis implemented in SimPlot ver3·5·1 with 500 bp window size and 20 bp step size^36^, Recombination Detection Program (RDP) ver4^37^ and jumping profile Hidden Markov Model (jpHMM)^38^. After getting the consensus breakpoint, fragment specific phylogenetic analysis was performed using ML-phylogenetic tree in RAxML.

### Evolutionary relationships inferred using family-joining

We used RAxML to estimate maximum likelihood distances under a GTR + Gamma model and constructed a phylogenetic tree using family-joining, as described recently^22^. The sequences were grouped into transmission clusters based on tree-based distances. Two sequences were considered to be in the same cluster if the corresponding tree-based distance was less than a pre-selected threshold. Transmission clusters were constructed for the *pol* tree (C_pol) and for the NFLG tree (C_NFLG), at distance thresholds of 0·02 subs/site, and 0·08 subs/site, respectively. The similarity of these two sets of clusters was calculated by the Jaccard index: Number of clusters in common/Number of distinct clusters present either in C_NFLG or in C_pol.

### Ethical considerations and data availability

The study was approved by regional ethics committee of Stockholm (2002/367; 2005/1167; 2007/1533; 2014/928–31/2) and all methods were performed in accordance with approved institutional guidelines. The patient identity was anonymised and delinked prior to analysis. The authors confirm that there are some restrictions on the data underlying the conclusions in the manuscript. The sequences that were analysed are representative of the entire country thereby, in principle, allow for the reconstruction of the transmission network^1^. Data are however available from the authors upon reasonable request and with permission of the steering committee of InfCare HIV. All the HIV-NFLG sequences generated in this study are available from GeneBank through accession numbers KP411823-KP411826, KP411828, KP411830-KP411845 and MF373124-MF373206.

## Electronic supplementary material

Supplementary Information
