# Supplementary material for: Recent increased identification and transmission of HIV-1 unique recombinant forms in Sweden
Source: Sci Rep. 2017 Jul 25;7:6371. doi: 10.1038/s41598-017-06860-2 (PMC5527090; doi:10.1038/s41598-017-06860-2)
Supplement: Supplementary file 1 — Supplementary Information [file 41598_2017_6860_MOESM1_ESM.doc]

**Recent increased identification and transmission of HIV-1 unique recombinant forms in Sweden**

Ujjwal Neogi ^1,*^, Abu Bakar Siddik ^1,#^, Prabhav Kalaghatgi^2^, Magnus Gisslén^3^, Göran Bratt^4^, Gaetano Marrone^5^, Anders Sonnerborg^1,6^

^1^Division of Clinical Microbiology, Department of Laboratory Medicine, Karolinska Institutet, Huddinge, Stockholm, Sweden

^2^Department of Computational Biology and Applied Algorithmics, Max Planck Institute for Informatics, Saarbrücken, Germany

^3^Department of Infectious Diseases, Sahlgrenska Academy, University of Gothenburg, Sweden

^4^Department of Infectious Diseases, South Hospital, Stockholm, Sweden

^5^Department of Public Health Sciences, Karolinska Institutet, Solna, Sweden

^6^Department of Medicine Huddinge, Unit of Infectious Diseases, Karolinska Institutet, Karolinska University Hospital, Stockholm, Sweden

**#Present Address:** Medical Microbiology Department, University of Manitoba, [727](https://www.facebook.com/) McDermot Ave, Winnipeg, MB R3E 3P5, Canada

**Table S1. Estimated time of transmission based on CD4^+^ T-cell decline trajectory model.**

| **PID** | **Lower Limit** | **Infection Year** | **Upper Limit** |
| --- | --- | --- | --- |
| Pt#019 | 1992 | 1993 | 1993 |
| Pt#001 | 1976 | 1980 | 1982 |
| Pt#016 | 1998 | 2000 | 2001 |
| Pt#003 | 1994 | 1996 | 1998 |
| Pt#005 | 1997 | 1999 | 2001 |
| Pt#004 | 1998 | 2001 | 2003 |
| Pt#009 | 1989 | 1993 | 1995 |
| Pt#007 | 2004 | 2005 | 2005 |
| Pt#008 | 1999 | 2001 | 2003 |
| Pt#093 | 1988 | 1992 | 1995 |
| Pt#010 | 1989 | 1992 | 1995 |
| Pt#015 | 1998 | 2001 | 2003 |
| Pt#013 | 1996 | 1999 | 2001 |
| Pt#022 | 2006 | 2006 | 2007 |
| Pt#023 | 1997 | 1999 | 2001 |
| Pt#020 | 1997 | 2000 | 2002 |
| Pt#024 | 1996 | 1999 | 2001 |
| Pt#027 | 1994 | 1998 | 2001 |
| Pt#028 | 2000 | 2002 | 2004 |
| Pt#032 | 2004 | 2007 | 2008 |
| Pt#080 | 1986 | 1989 | 1992 |
| Pt#006 | 1990 | 1994 | 1997 |
| Pt#012 | 1989 | 1992 | 1994 |
| Pt#096 | 1999 | 2000 | 2000 |
| Pt#048 | 2004 | 2004 | 2005 |
| Pt#063 | 1994 | 1998 | 2000 |
| Pt#002 | 1999 | 2001 | 2003 |
| Pt#025 | 2002 | 2004 | 2004 |
| Pt#095 | 1994 | 1998 | 2000 |
| Pt#014 | 1996 | 2000 | 2002 |
| Pt#021 | 2003 | 2006 | 2007 |
| Pt#085 | 1985 | 1988 | 1990 |
| Pt#035 | 1990 | 1994 | 1996 |
| Pt#029 | 1997 | 1998 | 2000 |
| Pt#062 | 2003 | 2003 | 2004 |
| Pt#018 | 1997 | 1999 | 2000 |
| Pt#026 | 2002 | 2006 | 2008 |
| Pt#030 | 1994 | 1996 | 1998 |
| Pt#031 | 2006 | 2008 | 2009 |
| Pt#040 | 2008 | 2009 | 2009 |
| Pt#039 | 2000 | 2002 | 2004 |
| Pt#038 | 1996 | 1998 | 1999 |
| Pt#066 | 1996 | 1998 | 2000 |
| Pt#036 | 2001 | 2003 | 2005 |
| Pt#097 | 1998 | 2002 | 2004 |
| Pt#060 | 2002 | 2005 | 2007 |
| Pt#050 | 2005 | 2007 | 2009 |
| Pt#045 | 2006 | 2009 | 2010 |
| Pt#061 | 2003 | 2006 | 2008 |
| Pt#044 | 2003 | 2005 | 2007 |
| Pt#057 | 2007 | 2009 | 2010 |
| Pt#075 | 2009 | 2010 | 2010 |
| Pt#047 | 1995 | 1997 | 1999 |
| Pt#055 | 2010 | 2010 | 2011 |
| Pt#046 | 2004 | 2007 | 2010 |
| Pt#052 | 1997 | 1999 | 2001 |
| Pt#054 | 2003 | 2007 | 2009 |
| Pt#053 | 2005 | 2008 | 2010 |
| Pt#051 | 2004 | 2006 | 2008 |
| Pt#051 | 2004 | 2006 | 2008 |
| Pt#072 | 1995 | 1998 | 2000 |
| Pt#073 | 2006 | 2009 | 2011 |
| Pt#067 | 1995 | 1998 | 2000 |
| Pt#068 | 2003 | 2006 | 2008 |
| Pt#064 | 2010 | 2011 | 2011 |
| Pt#076 | 2002 | 2004 | 2006 |
| Pt#077 | 2001 | 2005 | 2007 |
| Pt#065 | 2002 | 2004 | 2005 |
| Pt#081 | 2000 | 2003 | 2005 |
| Pt#082 | 1996 | 1999 | 2002 |
| Pt#086 | 2011 | 2012 | 2013 |
| Pt#090 | 1996 | 1999 | 2001 |
| Pt#056 | 2012 | 2012 | 2013 |
| Pt#087 | 2005 | 2007 | 2009 |
| Pt#078 | 2009 | 2011 | 2013 |
| Pt#079 | 1995 | 1998 | 2000 |
| Pt#084 | 2011 | 2012 | 2013 |
| Pt#088 | 2003 | 2005 | 2007 |
| Pt#094 | 2009 | 2011 | 2013 |


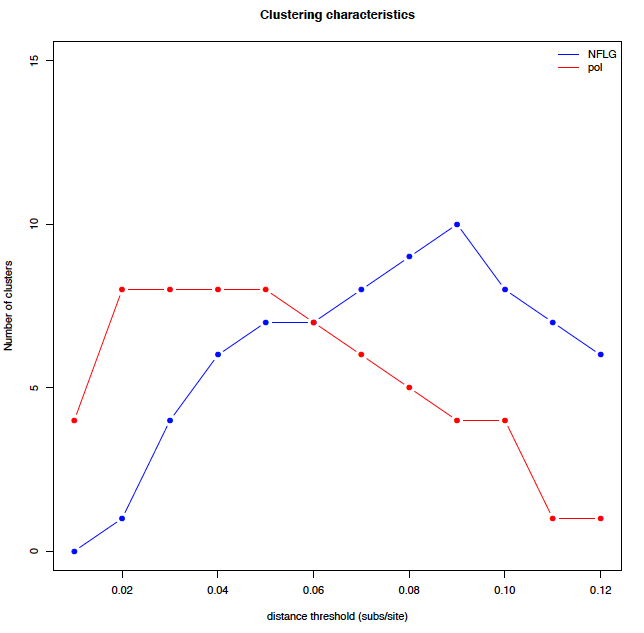


**Figure S2. Clustering statistics at different distance threshold**
